# Interference between Franck–Condon and Herzberg–Teller Terms in the Condensed-Phase Molecular Spectra of Metal-Based Tetrapyrrole Derivatives

**DOI:** 10.1021/acs.jpclett.2c01963

**Published:** 2022-08-05

**Authors:** Partha Pratim Roy, Sohang Kundu, Nancy Makri, Graham R. Fleming

**Affiliations:** † Department of Chemistry, University of California, Berkeley, Berkeley, California 94720, United States; ‡ Molecular Biophysics and Integrated Bioimaging Division, Lawrence Berkeley National Laboratory, Berkeley, California 94720, United States; § Kavli Energy Nanoscience Institute at Berkeley, Berkeley, California 94720, United States; ∥ Department of Chemistry, University of Illinois, Urbana, Illinois 61801, United States; ⊥ Department of Physics, University of Illinois, Urbana, Illinois 61801, United States; # Illinois Quantum Information Science & Technology Center, University of Illinois, Urbana, Illinois 61801, United States

## Abstract

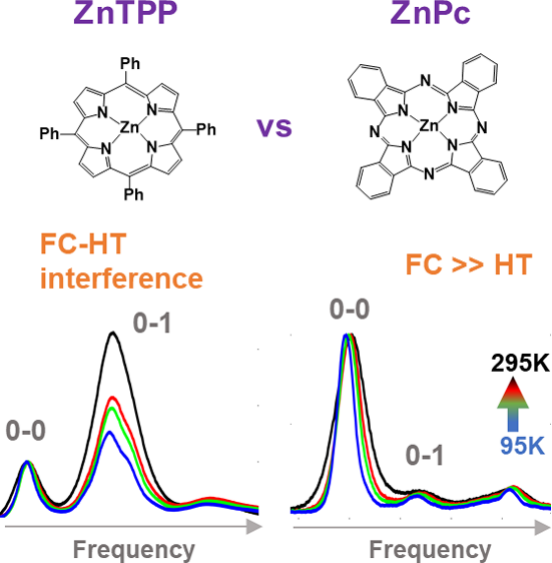

The commonly used Franck–Condon (FC) approximation is inadequate for explaining the electronic spectra of compounds that possess vibrations with substantial Herzberg–Teller (HT) couplings. Metal-based tetrapyrrole derivatives, which are ubiquitous natural pigments, often exhibit prominent HT activity. In this paper, we compare the condensed phase spectra of zinc–tetraphenylporphyrin (ZnTPP) and zinc–phthalocyanine (ZnPc), which exhibit vastly different spectral features in spite of sharing a common tetrapyrrole backbone. The absorption and emission spectra of ZnTPP are characterized by a lack of mirror symmetry and nontrivial temperature dependence. In contrast, mirror symmetry is restored, and the nontrivial temperature-dependent features disappear in ZnPc. We attribute these differences to FC–HT interference, which is less pronounced in ZnPc because of a larger FC component in the dipole moment that leads to FC-dominated transitions. A single minimalistic FC–HT vibronic model reproduces all the experimental spectral features of these molecules. These observations suggest that FC–HT interference is highly susceptible to chemical modification.

Tetrapyrrole rings form the structural backbone of most natural pigments that perform physiological functions in living organisms, such as energy, electron, or oxygen transport.^1−3^ Their large π-conjugated ring architecture provides high oscillator strengths and rigidity,^4−6^ fulfilling the basic criteria of building light harvesting molecular circuits.^7,8^ The photochemistry of tetrapyrrole derivatives, such as porphyrins and phthalocyanines, has been extensively characterized spectroscopically.^9−11^ Although ubiquitous in spectral analysis, the Franck–Condon (FC) approximation^12^ has been found inadequate for many porphyrin compounds.^13−16^ Rather, substantial first-order corrections to the transition dipole moment, known as Herzberg–Teller (HT)^17−19^ couplings, have been identified for certain vibrational modes.^20−23^ In sharp contrast, such effects are not observed in phthalocyanines.^24^ The prevalence of non-Condon effects in porphyrins and their susceptibility to the chemical modification of the tetrapyrrole ring offer exciting avenues of fine-tuning electronic and optical properties for materials design^6,25,26^ which may aid in the control of charge- and energy-transfer processes.

Despite early characterization, the interference of FC and HT terms in vibronic spectra, and especially its modulation by condensed phase environments and finite temperatures, still lack a clear understanding. Craig and Small^27^ pioneered the analysis of mirror-symmetry breaking in naphthalene spectra using a gas phase model at zero temperature. Similar reports exist for other aromatics such as benzene,^19^ anthracene,^28^ phenanthrene,^27^ pentacene,^29^ and porphyrin.^23^ Very few instances of the systematic inclusion of finite-temperature HT effects exist in the literature.^20,30,31^ Our recent work^32^ identified analytical symmetry arguments dictating the trends of FC–HT interference within the normal mode approximation and reported numerical calculations incorporating temperature dependence and homogeneous broadening.^32^ Non-Condon signatures were also recently found in excitation energy transfer^33,34^ as well as electron transfer^35,36^ dynamics. A theoretical study by Zhang et al.^33,34^ suggested that non-Condon vibronic coupling plays a central role in facilitating electronic–vibrational energy transfer and enhancing the quantum yield in contrast to FC vibronic coupling. Thus, in addition to traditional FC vibronic features, an intuitive understanding of HT signatures in molecular spectra is necessary to recognize the dynamical consequences of non-Condon effects.

In this paper, we investigate two metal-based tetrapyrrole derivatives, zinc–tetraphenylporphyrin (ZnTPP) and zinc–phthalocyanine (ZnPc), that share similar chemical structures but yield vastly different molecular spectra. Using a single minimalistic theoretical model, we show that the interference of FC and HT signatures can lead to mirror-asymmetric and nontrivially temperature-dependent spectral features in ZnTPP, which are entirely absent in ZnPc because of a dominant FC transition.

The absorption spectra of ZnTPP and ZnPc (Figure 1a) in a 3:1 mixture of diethyl ether and ethanol are shown in Figure 1b. This particular binary solvent mixture was chosen because it is suitable for solubilizing the tetrapyrrole derivatives as well as forming a good homogeneous glass at cryogenic temperature. The concentration of sample was kept low enough (0.08 mM for ZnTPP and 0.04 mM for ZnPc) to avoid any aggregation. The spectrum of ZnTPP exhibits a very intense band (ε_max_ = 5.7 × 10^5^ M^–1^ cm^–1^) at ∼23700 cm^–1^ and a weaker band (ε_max_ = 2.5 × 10^4^ M^–1^ cm^–1^) at λ_max_ ∼ 18000 cm^–1^. These bands are known as B (or Soret) and Q bands, respectively. In spite of similarities in the tetrapyrrole skeleton with ZnTPP, the absorption spectrum of ZnPc has a drastically different appearance. The B band (∼29450 cm^–1^) is much weaker (ε_max_ = 7.3 × 10^4^ M^–1^ cm^–1^) and blue-shifted compared to that of ZnTPP. On the other hand, the Q band (∼14900 cm^–1^) in ZnPc is ∼10 times more intense (ε_max_ = 2.97 × 10^5^ M^–1^ cm^–1^) and red-shifted compared to that in ZnTPP. A closer look at the Q band region shows a contrast in vibronic structure. While the Q (0–0) peak appears to be most intense in ZnPc, the 0–1 vibronic peak is the most intense in the Q band of ZnTPP, with a weak appearance of a Q (0–2) vibronic peak.

**Figure 1:**
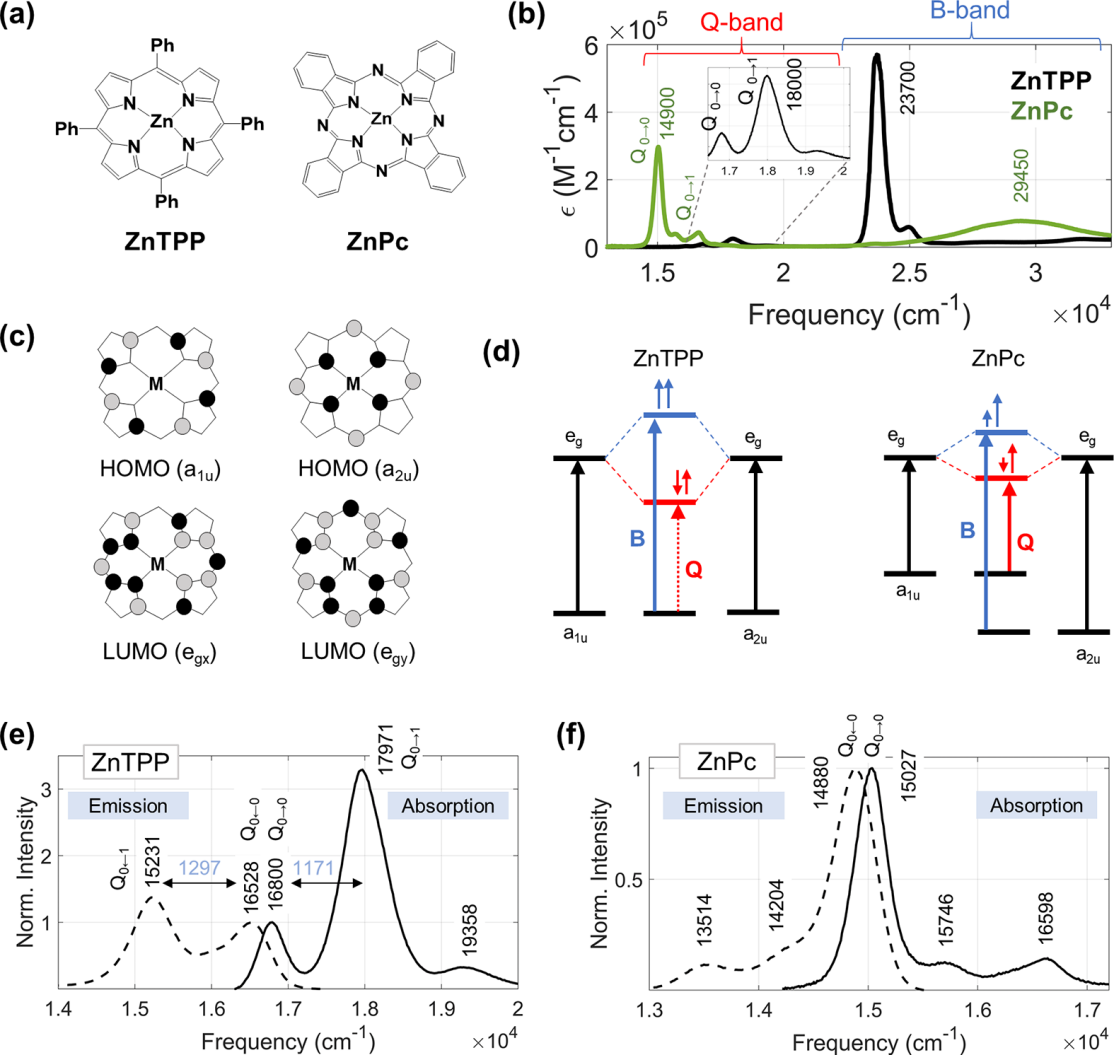
(a) Chemical structures of zinc–tetraphenylporphyrin (ZnTPP) and zinc–pthalocyanine (ZnPc). (b) UV–vis absorption spectra (unnormalized) of ZnTPP (black) and ZnPc (green) in a 3:1 mixture of diethyl ether and ethanol at room temperature. The B (or Soret) and Q bands are marked. (c) Illustration of HOMOs (a_1u_, a_2u_) and LUMOs (e_g*x*_, e_g*y*_) in the molecule with tetrapyrrole skeleton which has *D*_4*h*_ symmetry. The black and gray colors represent two opposite phases of the orbital. (d) Schematic energy diagram of Gouterman’s four-orbital model for ZnTPP (on left) in which a_1u_ and a_2u_ appear to be nearly degenerate and ZnPc (on right) in which the a_2u_ orbital is stabilized relative to its a_1u_ counterpart. The B and Q transitions are shown by blue and red arrows, respectively. The solid and dotted arrows represent allowed and forbidden transitions, respectively. The bottom panel shows the absorption (solid lines) and fluorescence emission (dashed lines) spectra of (e) ZnTPP and (f) ZnPc in a 3:1 mixture of diethyl ether and ethanol at room temperature. The spectra in (e) and (f) are normalized with respect to the Q (0–0) band.

The electronic transitions of tetrapyrrole macromolecules, which have *D*_4*h*_ symmetry, are usually understood by using Gouterman’s four-orbital model.^37^ The HOMOs have a_1u_ and a_2u_ symmetries whereas the LUMOs have e_g_ (*x*/*y*) symmetry, as illustrated in Figure 1c. In ZnTPP, the a_1u_ and a_2u_ orbitals are nearly degenerate. The large interaction between the two lowest energy orbital excitations (Figure 1d) causes the transition dipoles to add and form the intense B bands (B_*x*_ and B_*y*_); the excitations nearly cancel out (nearly forbidden electronic transition) to form weak Q bands (Q_*x*_ and Q_*y*_). In contrast, the degeneracy between the a_1u_ and a_2u_ orbitals is broken in ZnPc. The addition of the benzo group at the β position and substitution of a carbon atom with an electronegative nitrogen atom at the meso position of the tetrapyrrole ring cause relative stabilization of a_2u_ compared a_1u_ in ZnPc. Therefore, the interaction between the two lowest energy orbital excitations is reduced in ZnPc compared to that in ZnTPP (Figure 1d). This results in blue-shifted B bands and red-shifted Q bands in ZnPc. Because the transition dipoles do not cancel each other, the Q transition becomes more intense (allowed transition) with significant reduction in the intensity of the B bands as compared to ZnTPP. Note that degeneracy lifting between B_*x*_ and B_*y*_ transitions makes the B band appear broader in ZnPc compared to ZnTPP. Degeneracy lifting also causes the Q_*x*_ and Q_*y*_ peaks to split in ZnPc.

Intriguing differences are revealed by comparison of room temperature (∼295 K) absorption and fluorescence spectra of the two compounds (Figure 1e,f, where each spectrum is normalized with respect to the Q (0–0) peak maximum). ZnPc shows a near-perfect mirror symmetry relationship between absorption and emission with a dominant 0–0 band. In contrast, the mirror symmetry completely breaks down in ZnTPP with significant differences in the 0–1 band intensities. Moreover, for ZnTPP, the frequency gaps between the 0–0 and 0–1 peaks are substantially different in the absorption (1171 cm^–1^) and emission (1297 cm^–1^) spectra (a difference of 106 cm^–1^), creating an impression of significant differences between ground and excited potential energy curvatures.

Nontrivial behaviors also appear in the temperature dependence of the ZnTPP spectra (Figure 2a). With increase in temperature from 95 K to room temperature (∼295 K), three major changes are observed. First, in the emission spectrum, the 0–0 peak shows a large red-shift (∼150 cm^–1^), unlike the 0–1 peak, which shows only a small (∼20 cm^–1^) shift. Second, the disparity in energy gaps between 0−0 and 0–1 peaks in absorption and emission spectra is larger at lower temperatures compared to room temperature (265 cm^–1^ at 95 K vs 106 cm^–1^ at room temperature). Third, in the absorption spectrum, the relative intensity of the 0–1 band compared to the 0–0 band grows with increasing temperature. None of these three features appear in ZnPc (Figure 2b). Rather, a gradual (∼40–60 cm^–1^) and familiar blue-shift of all absorption peaks and a red-shift of emission peaks are observed with increase in temperature. These gradual small shifts are common and generally reflect the temperature dependence of the Stokes shift.^38,39^ They may also contain solvatochromic shifts resulting from the change in solvent polarity with temperature.^38^

**Figure 2:**
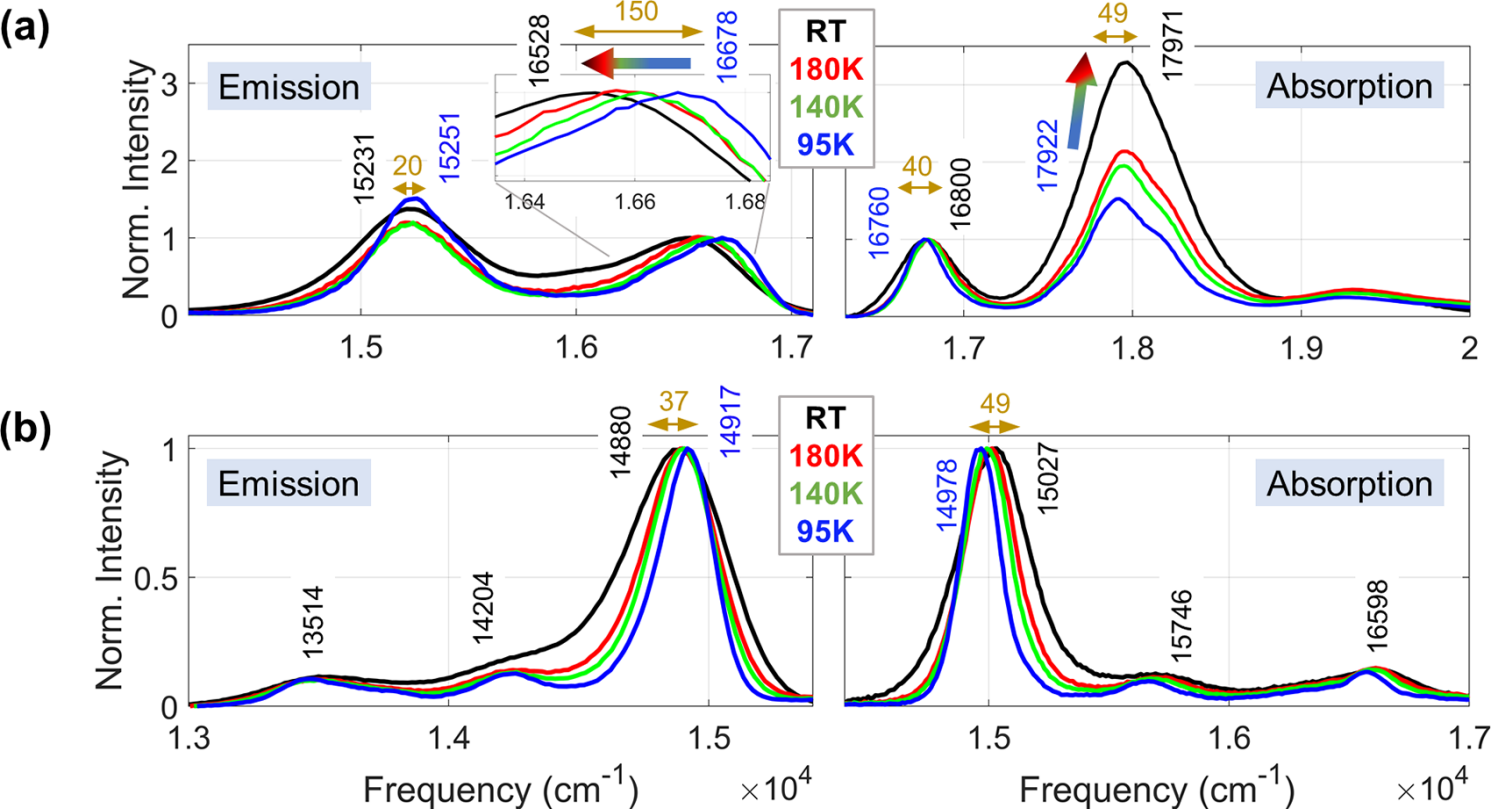
Temperature-dependent spectra. Absorption (right) and fluorescence emission (left) spectra of (a) ZnTPP and (b) ZnPc in a 3:1 mixture of diethyl ether and ethanol at room temperature (black), 180 K (red), 140 K (green), and 95 K (blue). Each spectrum was normalized with respect to the Q (0−0) band.

Gouterman’s four-orbital model excludes molecular vibrations and thus cannot rationalize differences in vibronic modulation of molecular spectra or its temperature dependence. To this end, we illustrate our simple model framework (Figure 3a).^32^ Expanding the transition dipole moment (in the direction of the applied electric field) along the normal mode coordinates **q** ≡ {*q*_*k*_} about a particular geometry {*q*_*k*_^eq^} of the molecule, we write
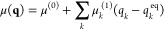
1.1where μ_*k*_^(1)^ is the first derivative of the dipole moment along the particular mode. The FC approximation involves a truncation at the zeroth-order, electronic dipole term μ^(0)^; thus, FC transitions follow Kasha’s rule,^41^ producing mirror-image symmetry between absorption and emission spectra.^38^ This approximation holds well for ZnPc (Figure 1f). However, a significant deviation from mirror symmetry and an intriguing asymmetric temperature dependence suggest a breakdown of the FC approximation for ZnTPP. The leading correction is the linear term in the normal mode coordinates, which is known as the HT contribution. Typically, when a transition is FC-allowed with a large oscillator strength, HT effects are overshadowed. When the FC transition is forbidden or only weakly allowed, HT-induced transitions may become dominant. Such transitions are often described as borrowing intensity from other strongly allowed FC transitions.^19^ Interference effects between FC and HT pathways can disrupt the mirror-image symmetry in linear spectra. Trends similar to those observed in ZnTPP were recovered in our recent FC–HT model study in various parameter regimes.^32^

**Figure 3:**
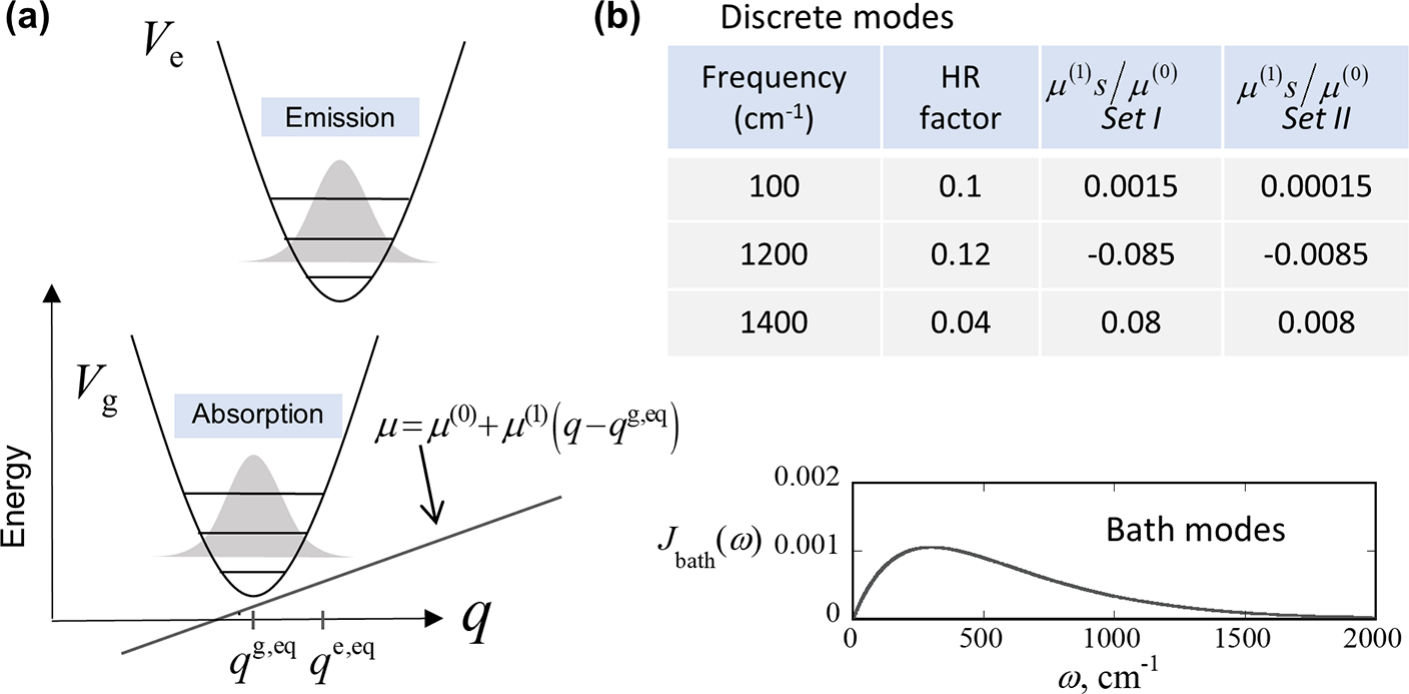
(a) Franck–Condon and Herzberg–Teller (FC–HT) model for transitions between two electronic potential surfaces (*V*_g_ and *V*_e_) coupled to a normal mode of vibration (*q*). (b) Vibrational parameters for the three discrete modes and the spectral density of the dissipative bath. The parameter *s* has dimensions of length. Sets I and II (obtained by a rescaling of set I dipole moments by a factor of 10) are used to model the spectra of ZnTPP and ZnPc, respectively.

The inadequacy of the FC approximation is common in porphyrin-based molecules.^13−16^ Significant intensity borrowing has been reported in Q transitions through HT vibronic mixing with the B transitions to produce the Q (0–1) band, which otherwise shows very weak FC activity with small Huang–Rhys (HR) factors caused by the rigid structure of the π-conjugated tetrapyrrole ring.^42−44^ The absorption spectra of monomeric metalloporphyrin complexes in the Q band region were recently discussed in detail.^23^ The Q band is derived from numerous modes in the low-frequency band and numerous 0 → 1 transitions (600–1500 cm^–1^) among HT active modes in the higher-frequency peak. On the basis of previous studies of porphyrin complexes and our previous FC–HT analysis,^32^ it is reasonable to hypothesize that the nontrivial features and temperature dependence in the ZnTPP spectra arise from FC–HT interferences. However, the HT pathway cannot be assumed to vanish in ZnPc, given the chemical similarity of the chromophores. The FC–HT model should thus not only account for the asymmetries and temperature dependence of the ZnTPP spectra but also (with physically motivated and reasonable modifications of parameters) recover the absence of HT effects in ZnPc. In what follows, we construct such a model. Note that our goal is not to simulate the spectra of ZnTPP or ZnPc but to capture the essential spectral features of both molecules by using a single minimalistic approach with the smallest possible number of explicit FC–HT active vibrational modes.

We use three discrete vibrations at 100, 1200, and 1400 cm^–1^ (see table in Figure 3b). The range of frequencies and the HT coupling parameters in “set I” are typical of metalloporphyrin complexes.^23^ Density functional theory (DFT) calculations have underestimated the HR factors of porphyrin vibrations.^20^ The chosen values of HR factors (0.05–0.15) are typical but larger than values reported based on DFT calculations. In addition, Figure 1 shows that (i) the intensity of the 0 → 0 peak in the Q region is over 10 times larger in ZnPc compared to ZnTPP and (ii) the intensity of the B band is nearly 8 times smaller in ZnPc compared to ZnTPP. For vibrational modes of moderate or low HR factor, the relatively small mixing of FC and HT pathways makes the 0–0 peak intensity mainly dependent on μ^(0)^, while a bulk of the 0–1 peak intensity arises from HT interactions alone. Moreover, the B band is known to determine the strength of HT coupling through intensity borrowing. Overall, the experiment therefore suggests at least an order of magnitude difference in the μ^(1)^*s*/μ^(0)^ values (parameter *s* has the dimension of length) of the two compounds which is further substantiated in the (iii) much larger ratio of 0–0 to 0–1 peak intensity in ZnPc compared to ZnTPP. Thus, we set the value of μ^(0)^ to be 10 times larger in “set II” (∼ZnPc) compared to its value in “set I” (∼ZnTPP), leaving all other parameters, including the relative interactions of the electronic states with the three discrete vibrations, unchanged. Last, we model the solvent and other normal mode vibrations of the complexes using a dissipative Ohmic bath^45^ that recently produced excellent agreement with pump–probe experiments for closely related cofacial porphyrin dimers.^46^ All model parameters are summarized in the table shown in Figure 3b.

Linear spectra (Figure 4a) calculated at different temperatures by using the three discrete modes with μ^(1)^*s*/μ^(0)^ values from “set I” of Figure 3b and the bath reproduce all experimental features for ZnTPP. Because the vibrations have low to moderate HR factors and HT couplings, the spectra are dominated by 0–0 and 0–1 bands for the discrete modes, with minor shoulders for the high-frequency 0–2 transitions. The observed broadening is entirely homogeneous and arises from the numerous combination excitations of the discrete modes and those of the dissipative bath. We note that the inclusion of a purely FC-active dissipative bath may nontrivially modulate the asymmetry of spectral bands because of FC–HT combination bands and asymmetric spectral shifts, as we discuss later. The severe mirror-image asymmetry in the 0–1 bands is recovered almost quantitatively through the interference of FC and HT terms in the 1200 and 1400 cm^–1^ modes. Our previous work^32^ showed that the direction of interference for each vibronic transition is reversed between absorption and emission spectra; that is, if a peak has amplified intensity through constructive interference in absorption, destructive interference and reduced intensity are observed in emission, and vice versa. Moreover, the vibronic transitions on either side of the 0–0 line are either all amplified or all diminished, depending on the relative signs of μ^(0)^ and μ_*k*_^(1)^.^32^ Because the HT couplings of the two high-frequency modes are opposite in sign, the 1200 cm^–1^ mode shows absorption dominance in the 0–1 peak, while the 1400 cm^–1^ peak is more pronounced in emission. As a result, the model spectra capture the feature of different energy gaps between the 0–0 and 0–1 peaks observed in the experiment.

**Figure 4:**
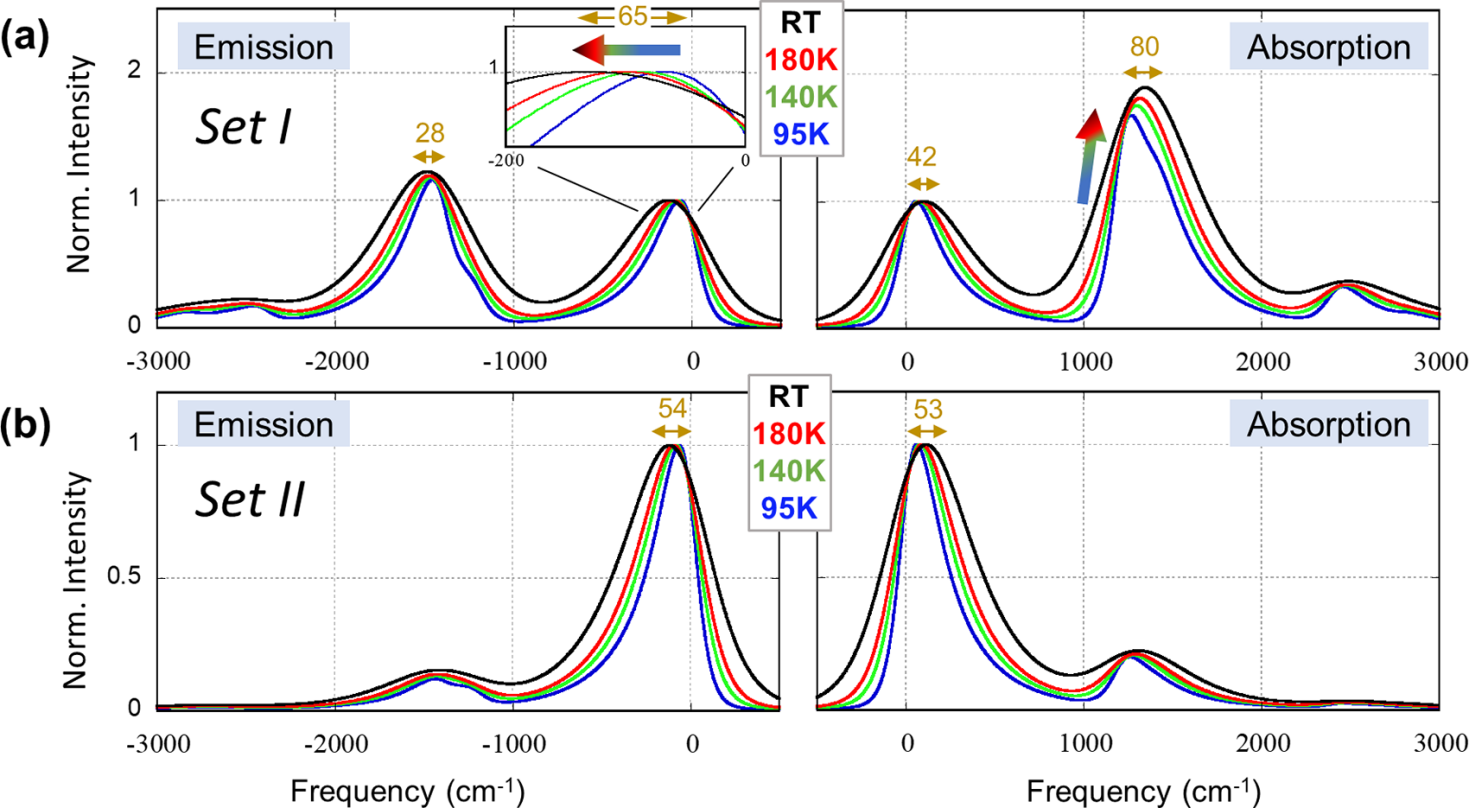
Temperature dependence (95–300 K) of theoretically calculated emission and absorption spectra are shown on left and right columns, respectively. All features in the experimental spectra of ZnTPP are reproduced (a) by using three discrete modes (with set I parameters in Figure 3b). Leaving everything the same but only increasing μ^(0)^ by a factor of 10 (see set II parameters in Figure 3b) produces spectra very similar to those of ZnPc in (b).

Typically, FC absorption and emission spectra exhibit blue- and red-shifts, respectively, with increase in temperature, which eventually (at sufficiently high temperatures) are given by the reorganization energy of the vibrational modes and the bath.^39,47,48^ However, when modes are FC–HT active, a change in temperature modifies the spectral asymmetry through differential interference patterns in transitions that originate from higher vibrational quanta.^32^ First, the disparity (Figure 2a) between thermal shifts of the 0–0 band in absorption (40 cm^–1^) and emission spectra (150 cm^–1^) hints at the presence of a low-frequency HT mode. Our model captures the asymmetric thermal shifts qualitatively through the inclusion of the 100 cm^–1^ mode with a very weak HT coupling, such that combinations between this mode and the Ohmic bath produce 42 and 65 cm^–1^ shifts in the theoretical absorption and emission spectra, respectively (Figure 4a). Modes in this frequency range (<100 cm^–1^) have been identified earlier in ZnTPP from low-temperature fluorescence experiments and characterized as torsional motions of the phenyl groups.^44^ A DFT calculation^20^ on free-base porphyrin found an HT-active mode at 150 cm^–1^. The 0–1 bands also exhibit different thermal shifts in absorption and emission spectra. The 80 and 28 cm^–1^ shifts observed in the theoretical absorption and emission spectra (Figure 4a) are in qualitative agreement with the shifts (49 and 20 cm^–1^) observed in the experiment (Figure 2a). This agreement illustrates the role of condensed phase environments in asymmetrically modulating FC–HT spectra.

Finally, our model also qualitatively captures the temperature-induced change in the intensity ratios of the 0–1 and 0–0 peaks in the absorption spectrum. This effect arises from two peaks (at 1200 and 1400 cm^–1^) that are of similar intensities in the absorption spectrum, such that the increase in homogeneous broadening with temperature leads to an overall increase in intensity of the 0–1 band. In contrast, the two peaks in emission have different intensities and therefore show no temperature dependence. Note that this effect is much more pronounced in the experimental spectra, possibly due to the involvement of many more FC–HT active modes^23^ in the 0–1 region. The ability of our simple model to qualitatively reproduce all trends observed in the experimental spectra of ZnTPP over a range of temperatures suggests that these nontrivial spectral features arise from FC–HT interference.

Next, we turn to the ZnPc spectra predicted by our model which involves a reasonable increase (factor of 10, set II) of the FC component of the dipole moment parameter which, as discussed earlier, is motivated by the relative intensities of the 0–0 and 0–1 peaks in the experimental spectra and leads to less prominent HT features. The spectra (Figure 4b) obtained with set II closely resemble those of ZnPc in Figure 2b. Because the vibrational frequencies of the two complexes are not expected to be identical, quantitative agreement along the frequency axis is not achievable by using the same modes. Nevertheless, we recover all the features and trends observed in the experiment: intense and almost symmetric 0–0 bands and slightly asymmetric but rather weak 0–1 bands. Moreover, the thermal shifts of the 0–0 band are now nearly identical in Figure 4b (53 and 54 cm^–1^) due to the quenching of FC–HT interference. These shifts are also in excellent agreement with the experimental shifts (49 and 37 cm^–1^, which are nearly identical considering our typical experimental error of 10 cm^–1^).

Overall, this study shows that a small modification in chemical structure can cause significant changes in the interference of FC and HT interactions and therefore result in vastly different molecular spectra. The condensed phase spectral signatures of ZnTPP and ZnPc as well as their contrasting temperature dependences are captured by a minimalistic FC–HT vibronic model. We find that the dominance of the FC-allowed pathway nearly quenches the dipole interferences in ZnPc, largely recovering the mirror symmetry relationship in ZnPc spectra consistent with the FC approximation. In contrast, a weak Q band transition that encourages significant vibronic intensity borrowing from the B band, leads to intricate patterns of FC–HT interferences that give rise to intriguing temperature-dependent characteristics in ZnTPP spectra. The intuitive understanding of FC–HT spectral features presented in this work will aid future studies of non-Condon vibronic coherence-mediated energy and charge transfer dynamics in coupled chromophore systems.

## Supporting Information Available

Transparent Peer Review report available (PDF)

## References

[ref1] Steinbach P. J.; Ansari A.; Berendzen J.; Braunstein D.; Chu K.; Cowen B. R.; Ehrenstein D.; Frauenfelder H.; Johnson J. B. Ligand Binding to Heme Proteins: Connections between Dynamics and Functions. Biochemistry 1991, 30, 3988–4001. 10.1021/bi00230a026. 2018767

[ref2] Auwärter W.; Écija D.; Klappenberger F.; Barth J. v. Porphyrins at Interfaces. Nat. Chem. 2015, 7, 105–120. 10.1038/nchem.2159. 25615664

[ref3] Mayo S. L.; Ellis W. R.; Crutchley R. J.; Gray H. B. Long-Range Electron Transfer in Heme Proteins. Science 1986, 233, 948–952. 10.1126/science.3016897. 3016897

[ref4] Fletcher J. T.; Therien M. J. Strongly Coupled Porphyrin Arrays Featuring Both π-Cofacial and Linear-π-Conjugative Interactions. Inorg. Chem. 2002, 41, 331–341. 10.1021/ic010871p. 11800622

[ref5] Lin V. S. Y.; DiMagno S. G.; Therien M. J. Highly Conjugated, Acetylenyl Bridged Porphyrins: New Models for Light-Harvesting Antenna Systems. Science 1994, 264 (5162), 1105–1111. 10.1126/science.8178169. 8178169

[ref6] Terazono Y.; Kodis G.; Chachisvilis M.; Cherry B. R.; Fournier M.; Moore A.; Moore T. A.; Gust D. Multiporphyrin Arrays with π-π Interchromophore Interactions. J. Am. Chem. Soc. 2015, 137 (1), 245–258. 10.1021/ja510267c. 25514369

[ref7] Ishizaki A.; Fleming G. R. Quantum Coherence in Photosynthetic Light Harvesting. Annual Review of Condensed Matter Physics 2012, 3, 333–361. 10.1146/annurev-conmatphys-020911-125126.

[ref8] Scholes G. D.; Fleming G. R.; Olaya-Castro A.; van Grondelle R. Lessons from Nature about Solar Light Harvesting. Nat. Chem. 2011, 3, 763–774. 10.1038/nchem.1145. 21941248

[ref9] Venkatesh Y.; Venkatesan M.; Ramakrishna B.; Bangal P. R. Ultrafast Time-Resolved Emission and Absorption Spectra of Meso-Pyridyl Porphyrins upon Soret Band Excitation Studied by Fluorescence Up-Conversion and Transient Absorption Spectroscopy. J. Phys. Chem. B 2016, 120, 9410–9421. 10.1021/acs.jpcb.6b05767. 27494567

[ref10] Kumble R.; Palese S.; Lin V. S. Y.; Therien M. J.; Hochstrasser R. M. Ultrafast Dynamics of Highly Conjugated Porphyrin Arrays. J. Am. Chem. Soc. 1998, 120, 11489–11498. 10.1021/ja981811u.

[ref11] Moretti L.; Kudisch B.; Terazono Y.; Moore A. L.; Moore T. A.; Gust D.; Cerullo G.; Scholes G. D.; Maiuri M. Ultrafast Dynamics of Nonrigid Zinc-Porphyrin Arrays Mimicking the Photosynthetic “Special Pair. J. Phys. Chem. Lett. 2020, 11 (9), 3443–3450. 10.1021/acs.jpclett.0c00856. 32290662

[ref12] Condon E. U. Nuclear Motions Associated with Electron Transitions in Diatomic Molecules. Phys. Rev. 1928, 32, 858–872. 10.1103/PhysRev.32.858.

[ref13] Kano H.; Saito T.; Kobayashi T. Observation of Herzberg-Teller-Type Wave Packet Motion in Porphyrin J-Aggregates Studied by Sub-5-Fs Spectroscopy. J. Phys. Chem. A 2002, 106, 3445–3453. 10.1021/jp012493f.

[ref14] Kee H. L.; Bhaumik J.; Diers J. R.; Mroz P.; Hamblin M. R.; Bocian D. F.; Lindsey J. S.; Holten D. Photophysical Characterization of Imidazolium-Substituted Pd(II), In(III), and Zn(II) Porphyrins as Photosensitizers for Photodynamic Therapy. J. Photochem. Photobiol., A 2008, 200 (2–3), 346–355. 10.1016/j.jphotochem.2008.08.006. PMC2615400 20016663

[ref15] Czernuszewicz R. S. Resonance Raman Spectroscopy of Metalloproteins Using CW Laser Excitation. Spectroscopic Methods and Analyses 2003, 345–374. 10.1385/0-89603-215-9:345. 21400146

[ref16] Spiro T. G.; Czernuszewicz R. S.; Li X. Y. Metalloporphyrin Structure and Dynamics from Resonance Raman Spectroscopy. Coord. Chem. Rev. 1990, 100, 541–571. 10.1016/0010-8545(90)85019-O.

[ref17] Ballhausen C. J.; Hansen A. E. Electronic Spectra. Annu. Rev. Phys. Chem. 1972, 23, 15–38. 10.1146/annurev.pc.23.100172.000311.

[ref18] Azumi T.; Matsuzaki K. Review Article What Does the Term “vibronic Coupling’ Mean?. Photochem. Photobiol. 1977, 25, 315–326. 10.1111/j.1751-1097.1977.tb06918.x.

[ref19] Struve W. S. Fundamentals of Molecular Spectroscopy, 22nd ed.; John Wiley & Sons: New York, 1989.

[ref20] Santoro F.; Lami A.; Improta R.; Bloino J.; Barone V. Effective Method for the Computation of Optical Spectra of Large Molecules at Finite Temperature Including the Duschinsky and Herzberg-Teller Effect: The Qx Band of Porphyrin as a Case Study. J. Chem. Phys. 2008, 128, 1–17. 10.1063/1.2929846. 18554017

[ref21] He R.; Li H.; Shen W.; Yang Q.; Li M. Vibronic Fine-Structure in the S 0 → S 1 Absorption Spectrum of Zinc Porphyrin: A Franck-Condon Simulation Incorporating Herzberg-Teller Theory and the Duschinsky Effect. J. Mol. Spectrosc. 2012, 275, 61–70. 10.1016/j.jms.2012.05.004.

[ref22] Minaev B.; Wang Y. H.; Wang C. K.; Luo Y.; Ågren H. Density Functional Theory Study of Vibronic Structure of the First Absorption Qx Band in Free-Base Porphin. Spectrochimica Acta Part A: Molecular and Biomolecular Spectroscopy 2006, 65 (2), 308–323. 10.1016/j.saa.2005.10.047. 16920011

[ref23] Pan Y.; Li L.; Qiu F.; Wei Y.; Hua W.; Tian G. On the Spectral Profile Change in the Q Band Absorption Spectra of Metalloporphyrins (Mg, Zn, and Pd): A First-Principles Study. J. Chem. Phys. 2019, 150 (16), 1–9. 10.1063/1.5090964. 31042882

[ref24] Isago H. Optical Spectra of Phthalocyanines and Related Compounds A Guide for Beginners; Springer: 2015.

[ref25] Tanaka T.; Osuka A. Conjugated Porphyrin Arrays: Synthesis, Properties and Applications for Functional Materials. Chem. Soc. Rev. 2015, 44 (4), 943–969. 10.1039/C3CS60443H. 24480993

[ref26] Osuka A.; Shimidzu H. Meso,Meso-Linked Porphyrin Arrays. Angewandte Chemie (International Edition in English) 1997, 36 (1–2), 135–137. 10.1002/anie.199701351.

[ref27] Craig D. F.; Small G. J. Totally Symmetric Vibronic Perturbations and the Phenanthrene 3400-Å Spectrum. J. Chem. Phys. 1969, 50 (9), 3827–3834. 10.1063/1.1671634.

[ref28] Zgierski M. Z. Herzberg-Teller Interactions and Spectra of Dimers: Stable Anthracene Dimer. J. Chem. Phys. 1973, 59, 3319 10.1063/1.1680476.

[ref29] Qian Y.; Li X.; Harutyunyan A. R.; Chen G.; Rao Y.; Chen H. Herzberg-Teller Effect on the Vibrationally Resolved Absorption Spectra of Single-Crystalline Pentacene at Finite Temperatures. J. Phys. Chem. A 2020, 124 (44), 9156–9165. 10.1021/acs.jpca.0c07896. 33103890

[ref30] Begušić T.; Vaníček J. On-the-Fly Ab Initio Semiclassical Evaluation of Vibronic Spectra at Finite Temperature. J. Chem. Phys. 2020, 153 (2), 024105 10.1063/5.0013677. 32668922

[ref31] Baiardi A.; Bloino J.; Barone V. General Time Dependent Approach to Vibronic Spectroscopy Including Franck-Condon, Herzberg-Teller, and Duschinsky Effects. J. Chem. Theory Comput. 2013, 9, 4097–4115. 10.1021/ct400450k. 26592403 PMC6485600

[ref32] Kundu S.; Roy P. P.; Fleming G. R.; Makri N. Franck-Condon and Herzberg-Teller Signatures in Molecular Absorption and Emission Spectra. J. Phys. Chem. B 2022, 126, 2899–2911. 10.1021/acs.jpcb.2c00846. 35389662

[ref33] Arsenault E. A.; Schile A. J.; Limmer D. T.; Fleming G. R. Vibronic Coupling in Energy Transfer Dynamics and Two-Dimensional Electronic-Vibrational Spectra. J. Chem. Phys. 2021, 155, 1–13. 10.1063/5.0056477. 34364357

[ref34] Zhang H. D.; Qiao Q.; Xu R. X.; Yan Y. Effects of Herzberg-Teller Vibronic Coupling on Coherent Excitation Energy Transfer. J. Chem. Phys. 2016, 145 (20), 204109 10.1063/1.4968031. 27908110

[ref35] Yoneda Y.; Sotome H.; Mathew R.; Lakshmanna Y. A.; Miyasaka H. Non-Condon Effect on Ultrafast Excited-State Intramolecular Proton Transfer. J. Phys. Chem. A 2020, 124 (2), 265–271. 10.1021/acs.jpca.9b09085. 31867968

[ref36] Kambhampati P.; Son D. H.; Kee T. W.; Barbara P. F. Solvent Effects on Vibrational Coherence and Ultrafast Reaction Dynamics in the Multicolor Pump-Probe Spectroscopy of Intervalence Electron Transfer. J. Phys. Chem. A 2000, 104 (46), 10637–10644. 10.1021/jp002549q.

[ref37] Fulton R. L.; Gouterman M. Vibronic Coupling. I. Mathematical Treatment for Two Electronic States. J. Chem. Phys. 1961, 1059, 35.

[ref38] Lakowicz J. R. Principles of Fluorescence Spectroscopy, 2nd ed.; Springer: 2006.

[ref39] de Jong M.; Seijo L.; Meijerink A.; Rabouw F. T. Resolving the Ambiguity in the Relation between Stokes Shift and Huang-Rhys Parameter. Phys. Chem. Chem. Phys. 2015, 17 (26), 16959–16969. 10.1039/C5CP02093J. 26062123

[ref41] Kasha M. Characterization of Electronic Transitions in Complex Molecules. Faraday Soc. Discussion 1950, 9, 14–19. 10.1039/df9500900014.

[ref42] Even U.; Jortner J. Isolated Ultracold Porphyrins in Supersonic. J. Chem. Phys. 1982, 77, 4391 10.1063/1.444440.

[ref43] Even U.; Magen J.; Jortner J.; Friedman J. Isolated Ultracold Porphyrins in Supersonic Expansions. II. Zn-Tetrabenzoporphyrin. J. Chem. Phys. 1982, 77, 4384–4390. 10.1063/1.444439.

[ref44] Even U.; Magen J.; Jortner J.; Friedman J.; Levanon H. Isolated Ultracold Porphyrins in Supersonic Expansions. I. Free-Base Tetraphenylporphyrin and Zn-Tetraphenylporphyrin. J. Chem. Phys. 1982, 77, 4374–4383. 10.1063/1.444438.

[ref45] Leggett A. J.; Chakravarty S.; Dorsey A. T.; Fisher M. P. A.; Garg A.; Zwerger W. Dynamics of the Dissipative Two-State System. Rev. Mod. Phys. 1987, 59, 1–85.

[ref46] Roy P. P.; Kundu S.; Valdiviezo J.; Bullard G.; Fletcher J. T.; Liu R.; Yang S.-J.; Zhang P.; Beratan D. N.; Therien M. J.; Makri N.; Fleming G. R. Synthetic Control of Exciton Dynamics in Bioinspired Cofacial Porphyrin Dimers. J. Am. Chem. Soc. 2022, 144, 6298–6310. 10.1021/jacs.1c12889. 35353523 PMC9011348

[ref47] Markham J. J. Interaction of Normal Modes with Electron Traps. Rev. Mod. Phys. 1959, 31, 956.

[ref48] Keil T. H. Shapes of Impurity Absorption Bands in Solids. Phys. Rev. 1965, 140, A601 10.1103/PhysRev.140.A601.

