# Supplementary material for: Interference between Franck–Condon and Herzberg–Teller Terms in the Condensed-Phase Molecular Spectra of Metal-Based Tetrapyrrole Derivatives
Source: J Phys Chem Lett. 2022 Aug 5;13(32):7413–9. doi: 10.1021/acs.jpclett.2c01963 (PMC9393888; doi:10.1021/acs.jpclett.2c01963)
Supplement: Supplementary file 1 — jz2c01963_si_001.pdf [file jz2c01963_si_001.pdf]

jz-2022-01963p.R1

Name: Peer Review Information for "Interference Between Franck-Condon and Herzberg-Teller Terms in the Condensed-Phase Molecular Spectra of Metal-Based Tetrapyrrole Derivatives"

First Round of Reviewer Comments

Reviewer: 1

Comments to the Author

1. What is the major advance reported in the paper?

The modest but important advance provided by this report is to extend the author's recent work theoretically investigating a model for the spectroscopically observable impact of interference between Frank-Condon and Herzberg-Teller (J. Phys. Chem. B 2022, 126, 2899−2911) to direct comparison with experimental measurements. The two systems were well selected to provide good contrast. This made it possible to demonstrate the quality of the model and ability to qualitatively explain deviations from the standard FC approximation as a function of temperature due to the HT first order correction.

2. What is the immediate significance of this advance?

In addition to providing new clarity for spectroscopic deviations in systems that fail to follow the simple and commonly assumed FC approximation, the simplicity of the model and qualitative insights offer significant advancement of a more intuitive understanding for basic electronic spectroscopy in common chromophores. In this regard, the work, and the clarity provided by the chosen example compounds, is in some ways a "textbook" example that will benefit many in a similar (although with more limited scope) way to grander examples such as Kasha's rule. There will be a gain for many in their basic understanding of non-Condon effects that is generally lacking.

3. Technical suggestions

Nothing significant. The manuscript is clear and well written. The questions are clear and the arguments are well supported and justified.

Not necessary, but a comment to explain the choice of the diethyl ether : ethanol solvent (solubility one might assume?) would be a nice addition. It could also be useful to mention that the same binary solvent was used in ref 44, which provided the justification for the spectrum of the ohmic bath used.

Reviewer: 2

Comments to the Author

In this paper the authors compare the linear absorption and emission spectra of ZnTPP and ZnPc. The authors perform a temperature-dependent study of linear spectra and interpret the spectral changes using a FC-HT model that was introduced by the authors in their previous work. The authors show that the FC-HT model can qualitatively reproduce the overall spectral changes, including temperature dependent changes, in absorption and emission of ZnTPP and ZnPc. To model the spectra the authors included three vibrational modes coupled to the electronic states. To describe the different molecules the authors alter the magnitude of the FC contribution to the transition dipole moment, where the FC contribution is 10 times larger in ZnPc. As ZnTPP is assumed to have larger HT coupling based on the linear absorption spectra, the contribution from the FC term was assumed to be smaller than ZnPC. Altering these parameters, the model is able to reproduce the experimental observations. The authors conclude that the structural changes between ZnTPP and ZnPC change the interference between FC-HT active modes leading to the differences in the absorption and emission spectra.

This work shows that structural changes can lead to a change in HT activity which can impact the linear spectra of molecules through FC-HT interference. The spectral changes can be understood through a simple model which could provide more intuition into how different vibrational couplings imprint on electronic spectra. The paper will be of interest to researchers in the field and I recommend the paper for publication.

Below I offer some suggestions that may help to improve the paper.

1. The authors use the same model to describe the linear spectra of ZnTPP and ZnPc, where the FC contribution is much stronger for ZnPc. The two molecules ZnPc and ZnTPP studied, have different molecule structures where the phenyl groups on ZnPc increase conjugation of the ring system. Given the structural differences, one may expect different vibrational modes to be coupled to the electronic states. The authors should further justify their choices in using the same vibrational modes for both molecules.

In addition, the authors note that the HT couplings of the 1200 cm-1 and 1400 cm-1 mode are opposite in sign for the ZnTPP and ZnPc. How was this determined – through a comparison of the general spectral features? If so, it seems it would be difficult to evaluate the HT coupling for ZnPc where the FC is weighted more. Another way to ask this question is if the spectrum of ZnPc can be modeled without the HT coupling, or if the HT coupling is included how sensitive is the simulated spectra to changes in the HT coupling.

2. On page 6, I think that the authors have the wrong citations for Gouterman’s four orbital model. The authors cite the Fulton and Gouterman papers for a vibronically coupled dimer. These references should be corrected.

3. The authors should report the concentration of ZnTPP and ZnPc used for the measurements. Aggregation could lead to similar spectral changes – how was aggregation ruled out?

4. A parameter s is introduced in the table in Fig. 3b. The parameter should be defined in the text.

Author's Response to Peer Review Comments:

Dear Editor,

Please find attached a revised version of our manuscript "Interference between Franck-Condon and Herzberg-Teller Terms in the Condensed-phase Molecular Spectra of Metal-based Tetrapyrrole Derivatives" jz-2022-01963p by Roy et al, for publication in Journal of Physical Chemistry Letter. We thank the reviewers for critical reading of the manuscript. We have accepted and addressed all changes suggested by the reviewers.
In the following sentences we address the remarks of each referee individually.

Response to Reviewer 1
1. What is the major advance reported in the paper?
The modest but important advance provided by this report is to extend the author’s recent work theoretically investigating a model for the spectroscopically observable impact of interference between Frank-Condon and Herzberg-Teller (J. Phys. Chem. B 2022, 126, 2899−2911) to direct comparison with experimental measurements. The two systems were well selected to provide good contrast. This made it possible to demonstrate the quality of the model and ability to qualitatively explain deviations from the standard FC approximation as a function of temperature due to the HT first order correction.
2. What is the immediate significance of this advance?
In addition to providing new clarity for spectroscopic deviations in systems that fail to follow the simple and commonly assumed FC approximation, the simplicity of the model and qualitative insights offer significant advancement of a more intuitive understanding for basic electronic spectroscopy in common chromophores. In this regard, the work, and the clarity provided by the chosen example compounds, is in some ways a “textbook” example that will benefit many in a similar (although with more limited scope) way to grander examples such as

Kasha's rule. There will be a gain for many in their basic understanding of non-Condon effects that is generally lacking.

3. Technical suggestions

Nothing significant. The manuscript is clear and well written. The questions are clear and the arguments are well supported and justified.

Not necessary, but a comment to explain the choice of the diethyl ether: ethanol solvent (solubility one might assume?) would be a nice addition. It could also be useful to mention that the same binary solvent was used in ref 44, which provided the justification for the spectrum of the ohmic bath used.

Author reply: We thank the reviewer for recognizing the significance of the paper and for the technical suggestion. The reviewer guessed it right that we used diethyl ether because it is suitable to solubilize tetrapyrrole derivatives. We use binary solvent (mixture of diethyl ether and ethanol with 3:1 ratio) to form a good homogenous glass at cryogenic temperature. We added a sentence on page 5 to clarify this to the readers: "This particular binary solvent mixture was chosen because it is suitable for solubilizing the tetrapyrrole derivatives and forming a good homogenous glass at cryogenic temperature."

Responses to Reviewer 2

1. The authors use the same model to describe the linear spectra of ZnTPP and ZnPc, where the FC contribution is much stronger for ZnPc. The two molecules ZnPc and ZnTPP studied, have different molecule structures where the phenyl groups on ZnPc increase conjugation of the ring system. Given the structural differences, one may expect different vibrational modes to be coupled to the electronic states. The authors should further justify their choices in using the same vibrational modes for both molecules.

In addition, the authors note that the HT couplings of the 1200 cm-1 and 1400 cm-1 mode are opposite in sign for the ZnTPP and ZnPc. How was this determined – through a comparison of the general spectral features? If so, it seems it would be difficult to evaluate the HT coupling for ZnPc where the FC is weighted more. Another way to ask this question is if the spectrum of ZnPc can be modelled without the HT coupling, or if the HT coupling is included how sensitive is the simulated spectra to changes in the HT coupling.

Author reply: We thank the reviewer for raising this question. The reviewer is right in pointing out that the increase in conjugation from ZnTPP to ZnPc is expected to change the vibrational modes. However, the localized high (>1000 cm-1) frequency modes are usually much less affected compared to delocalized low (<300 cm-1) frequency modes by the change in conjugation. Although we don't rule out the changes in vibrations from ZnTPP to ZnTPP, we chose the vibrations to be the same for both in our model because our goal was not to simulate

the spectra but to capture the essential spectral features of both molecules using a single minimalistic approach (as stated on page 11) in order to understand the sole effect of HT coupling on spectral features and its non-trivial temperature dependence.
Santoro et al (reference 20) reported a number of HT active high frequency modes different signs of µ1. We chose the opposite sign of µ1 for the 1200 and 1400 cm-1 modes in set I to model the contrasting behaviour observed in the experimental temperature dependent absorption and emission spectra of ZnTPP. It is indeed difficult to quantitatively estimate the change in HT coupling from ZnTPP to ZnPc. We assume the µ1 not to be significantly different given the structural similarity between the two compounds. This is why we changed the weight of FC activity (compared to HT coupling) while keeping the other parameters fixed to reproduce the ZnPc spectral features.

2. On page 6, I think that the authors have the wrong citations for Gouterman's four orbital model. The authors cite the Fulton and Gouterman papers for a vibronically coupled dimer. These references should be corrected.
Author reply: We apologize for this typo. We have removed the citation of ref 38 (vibronically couple dimer paper) and only kept the citation of ref 37 (Gouterman's four orbital model) on page 6.

3. The authors should report the concentration of ZnTPP and ZnPc used for the measurements. Aggregation could lead to similar spectral changes – how was aggregation ruled out?
Author reply: The concentrations of ZnTPP and ZnPc were 0.08 mM and 0.04 mM, which are sufficiently dilute to avoid the aggregation. We did not observe any concertation dependent change in spectral features in the concentration range from 0.01 to 0.2 mM for either molecule. We added the concentration values on page 6: "The concentration of sample was kept low enough (0.08 mM for ZnTPP and 0.04 mM for ZnPc) to avoid aggregation."

4. A parameter s is introduced in the table in Fig. 3b. The parameter should be defined in the text.
Author reply: We added a sentence on page 12: (parameter s has the dimension of length).
